# Medical treatment of Cushing’s disease with concurrent diabetes mellitus

**DOI:** 10.3389/fendo.2023.1174119

**Published:** 2023-04-17

**Authors:** Anna Mehlich, Marek Bolanowski, Dawid Mehlich, Przemysław Witek

**Affiliations:** ^1^ Department of Internal Medicine, Endocrinology and Diabetes, Medical University of Warsaw, Warsaw, Poland; ^2^ Chair and Department of Endocrinology, Diabetes, and Isotope Treatment, Wroclaw Medical University, Wroclaw, Poland; ^3^ Laboratory of Molecular OncoSignalling, International Institute of Molecular Mechanisms and Machines (IMol) Polish Academy of Sciences, Warsaw, Poland; ^4^ Doctoral School of Medical University of Warsaw, Medical University of Warsaw, Warsaw, Poland; ^5^ Laboratory of Experimental Medicine, Medical University of Warsaw, Warsaw, Poland

**Keywords:** Cushing’s disease, diabetes mellitus, insulin resistance, medical therapies, metabolic comorbidities, glucose metabolism

## Abstract

Cushing’s disease (CD) is a severe endocrine disorder characterized by chronic hypercortisolaemia secondary to an overproduction of adrenocorticotropic hormone (ACTH) by a pituitary adenoma. Cortisol excess impairs normal glucose homeostasis through many pathophysiological mechanisms. The varying degrees of glucose intolerance, including impaired fasting glucose, impaired glucose tolerance, and Diabetes Mellitus (DM) are commonly observed in patients with CD and contribute to significant morbidity and mortality. Although definitive surgical treatment of ACTH-secreting tumors remains the most effective therapy to control both cortisol levels and glucose metabolism, nearly one-third of patients present with persistent or recurrent disease and require additional treatments. In recent years, several medical therapies demonstrated prominent clinical efficacy in the management of patients with CD for whom surgery was non-curative or for those who are ineligible to undergo surgical treatment. Cortisol-lowering medications may have different effects on glucose metabolism, partially independent of their role in normalizing hypercortisolaemia. The expanding therapeutic landscape offers new opportunities for the tailored therapy of patients with CD who present with glucose intolerance or DM, however, additional clinical studies are needed to determine the optimal management strategies. In this article, we discuss the pathophysiology of impaired glucose metabolism caused by cortisol excess and review the clinical efficacy of medical therapies of CD, with particular emphasis on their effects on glucose homeostasis.

## Introduction

Cushing’s syndrome (CS) is a severe endocrine disorder caused by chronic exposure to excess glucocorticoids (GCs), which can be from exogenous or endogenous origin. Endogenous CS is further classified as adrenocorticotropin (ACTH)-dependent Cushing’s syndrome (80% of all cases) and ACTH-independent (1). Cushing’s disease (CD), which results from an uncontrolled adrenocorticotrophic hormone (ACTH) secretion from a pituitary tumor, is the most common form of ACTH-dependent CS. The prevalence of CD is estimated to be nearly 40 cases per million, and the incidence of CD ranges from 1.2 to 2.4 per million per year, although these figures might be underestimated due to undiagnosed patients who experience mild or variable disease course (2–5).

The untreated CD is associated with excessive mortality and morbidity as well as decreased quality of life (6, 7). The clinical picture of CD consists of weight gain, central obesity with facial fat redistribution, skin changes, depression, cognitive impairment, susceptibility to infections, menstrual irregularities in women, and decreased libido in men (8, 9). Patients with CD commonly develop multiple systemic and metabolic complications, including insulin resistance, prediabetes, diabetes mellitus (DM), dyslipidemia, hypertension, and hypercoagulability. The prevalence of DM in patients with CD ranges from 20 to 50%, while 10-30% of patients have impaired glucose tolerance. Collectively, glucose metabolism abnormalities are observed in approximately 70% of patients with CD and represent one of the most common complications of the disease (3–8, 10–13). Diagnosis of prediabetes and diabetes in patients with CS is the same as in the general population and is based on measurements of fasting plasma glucose, HbA1c, and plasma glucose values under an oral glucose tolerance test (OGTT) (14). However, it should be noted that the most important metabolic effects of GCs excess occur during the post-prandial period, and a substantial proportion of patients with CS may present with normal fasting glucose (15). Thus, the OGTT is considered a gold standard in diagnosing glucose metabolism abnormalities in this population. An approach based on OGTT can also be applied to evaluate pancreatic beta cells dysfunction in patients with chronic hypercortisolaemia (16).

Cortisol excess plays a major role in the pathogenesis of impaired glucose tolerance, and the severity of hypercortisolaemia generally correlates with glucose metabolism derangements. Moreover, genetic and environmental factors significantly contribute to the impairment of glucose tolerance and account for the interindividual susceptibility to disturbed glucose homeostasis induced by the cortisol excess (13, 17, 18). Definitive surgical treatment of ACTH-secreting tumors is the first-line therapy for patients with CD, including those with concurrent DM (19). Nevertheless, medical therapies have gained a significant role in the treatment of CD patients for whom surgical treatment of pituitary tumors was unsuccessful or for those that are ineligible to undergo surgery (19, 20). In this article, we review the pathophysiology of glucose metabolism abnormalities caused by hypercortisolaemia and discuss the clinical effectiveness of medical treatments in the management of CD patients with DM.

## Pathophysiology of glucocorticoid-induced insulin resistance and diabetes mellitus in patients with Cushing’s disease

In humans, glucocorticoid hormones are represented primarily by cortisol produced in the adrenal glands. In rodents, corticosterone is the main adrenal cortex hormone, and thus it is commonly used in experimental studies to investigate GCs functions in animal models. Additionally, several synthetic GCs are frequently used in preclinical studies, including prednisone, methylprednisolone, and dexamethasone (21).

The release of ACTH and endogenous GCs is under the control of circadian rhythm and stress. In humans under physiological conditions, blood GCs levels peak in the early morning, decline throughout the daytime, and nadir around midnight (22). Glucocorticoid hormones play a major role in the physiological regulation of energy homeostasis. In the postprandial period, GCs counteract the anabolic effects of insulin and provide the main substrates for oxidative metabolism, including glucose, amino acids, and fatty acids, through the stimulation of gluconeogenesis, lipolysis, and proteolysis as well as inhibition of glycogen synthesis.

**Figure 1:**
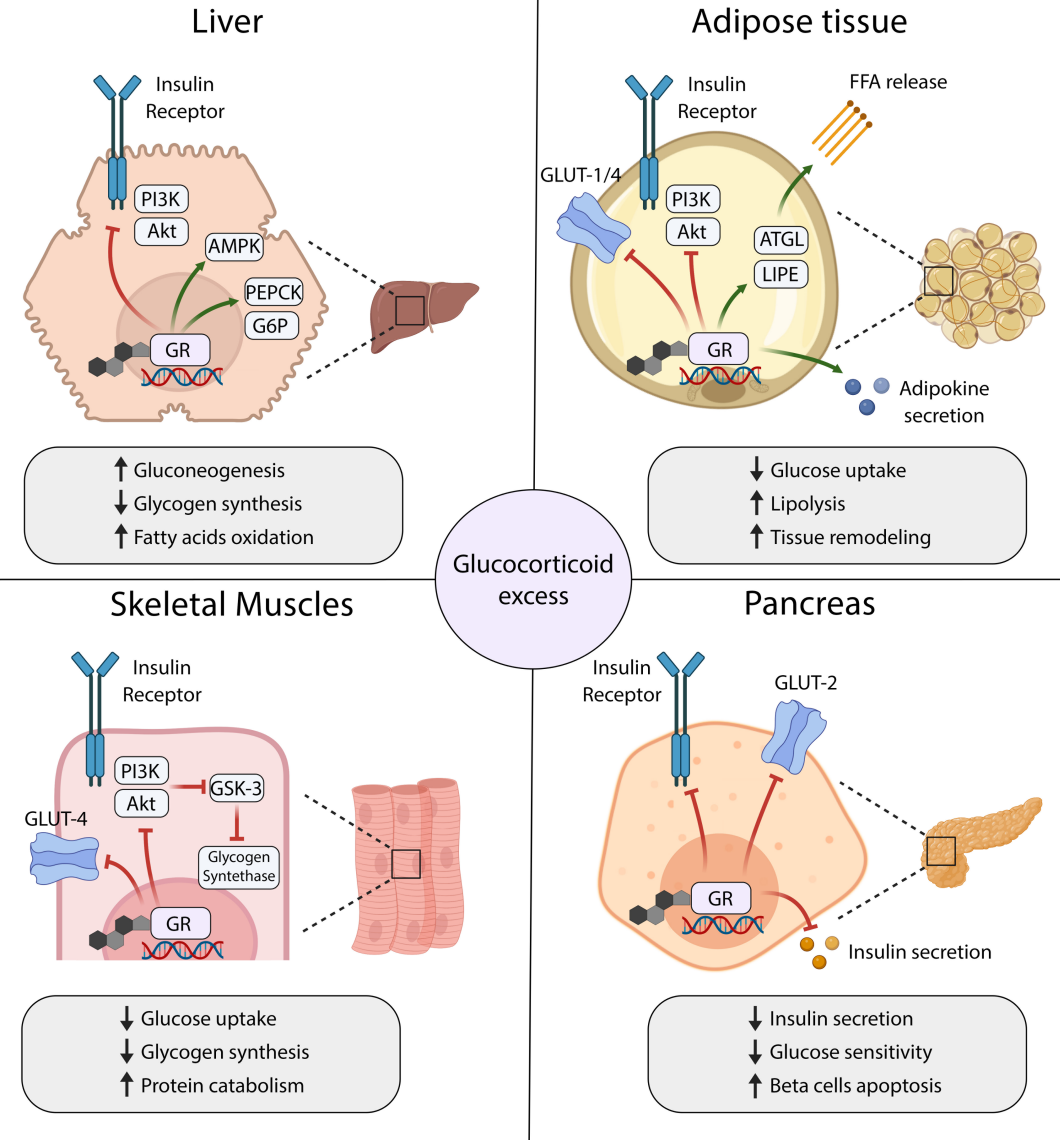
Mechanisms of glucocorticoid-induced insulin resistance and diabetes mellitus in main organs that control glucose homeostasis. GR, Glucocorticoid Receptor; FFA, free fatty acids; ATGL, Adipose triglyceride lipase; LIPE, lipase, hormone-sensitive.

In patients with CS, chronic exposure to cortisol leads to insulin resistance, hyperglycemia, derangements of lipid metabolism, and altered body composition. In addition to GCs excess, the impairment of circadian GCs secretion also contributes to metabolic abnormalities (23). Chronic and uncontrolled hypercortisolaemia profoundly affects the physiological functions of key metabolic organs, including the liver, muscles, adipose tissue, and pancreas (17, 24) (**Figure 1**).

In the liver, GCs regulate glucose metabolism both directly through the activation of gluconeogenesis and indirectly through the stimulation of the hepatic insulin resistance (17). Glucocorticoids promote the transcription of genes encoding key enzymes in the gluconeogenic pathway, including Phosphoenolpyruvate Carboxykinase-1 (PEPCK) and Glucose-6-phosphate dehydrogenase (G6PD), to increase glucose production in the liver (25–27). At the same time, GCs attenuate the signal transduction downstream of insulin receptor (IR) and decrease insulin sensitivity in hepatocytes. Mechanistically, GCs activate the transcription of several genes whose products inhibit the PI3K/Akt/mTOR signaling pathway, a major downstream effector of the IR cascade (28–30). Furthermore, GCs facilitate the activation of Adenosine monophosphate-dependent kinase (AMPK), which in turn switches on the catabolic pathways, including fatty acids oxidation. Thus, GCs excess leads to hyperglycemia and may contribute to progressive liver steatosis in patients with hypercortisolaemia (31).

Skeletal muscles are major sites for insulin-stimulated glucose uptake, utilization, and storage in the form of glycogen. They also form a reservoir of amino acids, which can be used as substrates for glucose production in the process of gluconeogenesis. Because of their key roles in regulating glucose metabolism, skeletal muscles are also considered the primary driver of whole-body insulin resistance in patients with hypercortisolaemia (32). In skeletal muscles, GCs negatively regulate the signaling cascades downstream of IR, mainly through PI3K/Akt/mTOR pathway (29, 33, 34). These actions result in reduced membrane translocation of Glucose transporter type 4 (GLUT-4), and consequently inhibition of the insulin-stimulated glucose uptake (35). Furthermore, low activity of Akt kinase stimulates Glycogen synthase kinase 3 (GSK-3), which acts as a major negative regulator of the Glycogen synthase (34). Thus, GCs decrease glycogen synthesis in skeletal muscle cells. They also appear to have a permissive role in the catecholamine-dependent glycogenolysis (36).

The adipose tissue controls glucose homeostasis through the processes of lipogenesis (*de novo* synthesis of fatty acids from glucose), lipolysis, and secretion of endocrine factors that affect insulin sensitivity in many tissues. GCs seem to play a pivotal role in regulating the metabolism, differentiation, and distribution of adipose tissue. Long-term hypercortisolaemia leads to visceral accumulation of fat tissue and obesity, commonly observed in patients with CD (37). In mature adipocytes, GCs stimulate lipolysis by increasing the expression of lipases and induce insulin resistance by inhibiting signaling pathways downstream of IR. Consequently, GCs excess leads to the downregulation of glucose transporters and decreased glucose uptake by adipocytes, as well as increased release of free fatty acids (35, 38). Moreover, GCs may stimulate the secretion of adipokines that contribute to the remodeling of adipose tissue and further augment the insulin resistance (39, 40).

Glucocorticoids also directly affect the function of pancreatic beta cells and insulin secretion, however, the exact mechanisms underlying this phenomenon remain poorly understood. *In vitro*, GCs were found to decrease the viability of beta cells, downregulate Glucose transporter type 2 (GLUT-2) expression, and impair insulin biosynthesis and release (41–44). Intriguingly, several studies in animal models showed beta cells hyperplasia and transient hyperinsulinemia in response to GCs treatment, which could represent an adaptive response to maintain euglycemia (45, 46). In humans, both impaired insulin secretion and hyperinsulinemia were reported in various clinical studies that evaluated pancreatic response to acute or short-term treatment with GCs (47–54). The contradictory results between the studies likely reflect the significant differences in study design, type and duration of the treatment, route of GCs agent administration, and different accompanying clinical procedures. Nonetheless, chronic exposure to GCs appears to induce beta cell dysfunction due to the inhibition of insulin secretion and induction of apoptosis, and these effects presumably contribute to the development of glucose metabolism abnormalities in patients with CS (13, 15, 47).

In addition to the mechanisms described above, several other organs and tissues are likely involved in the pathogenesis of glucocorticoid-induced insulin resistance, including bone, gut, and brain (27). The studies in the animal models showed that GCs-induced metabolic syndrome-like phenotype with central obesity and insulin resistance persists for a long time after hypercortisolaemia remission (55). These results correspond to clinical data from patients with CS, which demonstrated increased cardiometabolic risk along with persistent abdominal fat accumulation and insulin resistance even after the remission of the disease (56). The mechanisms underlying long-lasting metabolic derangements after disease remission in humans are poorly understood and likely involve a complex interplay between hormonal deficiencies and persistent harm induced by GCs in the target tissues.

## Medical therapies in the treatment of Cushing’s disease and concurrent diabetes mellitus

**Figure 2:**
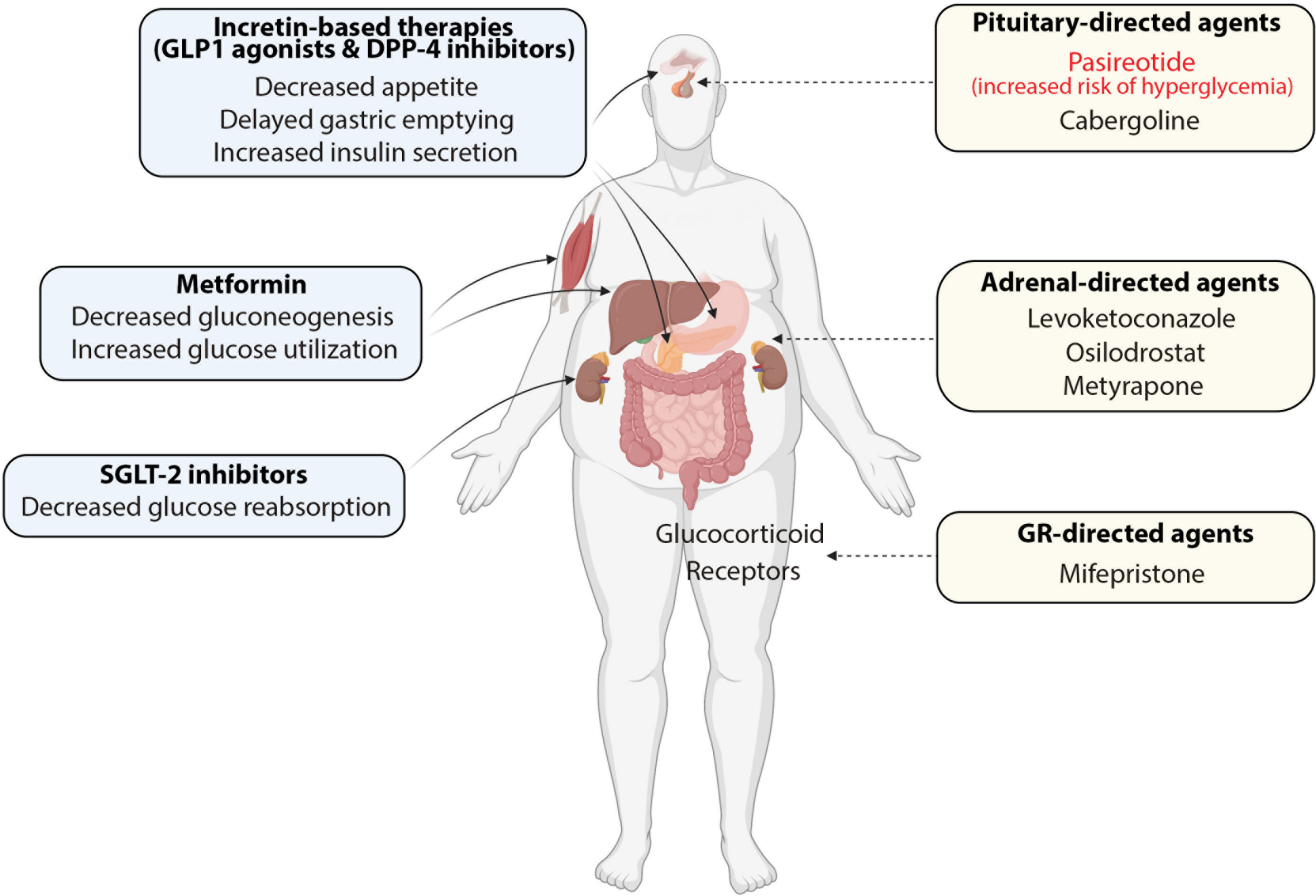
Combinations of antidiabetic medications (blue boxes) and cortisol-lowering agents (yellow boxes) in the treatment of patients with Cushing’s disease and concurrent diabetes mellitus.

The major therapeutic goal in patients with CD is to decrease endogenous cortisol levels. Surgical removal of pituitary adenoma is considered the first-line therapeutic option for patients with CD. Following surgery, remission of hypercortisolism is observed in 70-90% of patients, and the risk of disease recurrence ranges from 10-20%. Nevertheless, long-term failure of the surgical procedure is observed in one-third of patients, who require additional treatments (12, 57, 58). Thus, second-line therapies, such as radiotherapy, bilateral adrenalectomy, and medical therapy, should be considered in patients who relapsed after initial surgery or were reluctant or ineligible to undergo the surgical procedure (19). In the past decades, medical therapy has emerged as a particularly attractive adjunctive treatment strategy in patients with CD, mainly due to successful drug development efforts, as well as a growing body of clinical evidence indicating the efficacy and safety of old and new medications (20). Currently, three major drug categories are used in CD treatment, including 1) pituitary-directed drugs represented by pasireotide and cabergoline; 2) adrenal-directed drugs, represented by ketoconazole, levoketoconazole, metyrapone, mitotane, and osilodrostat; 3) Glucocorticoid Receptor (GR)-directed drugs, mainly represented by mifepristone and investigational drug relacorilant (**Figure 2**).

Adrenal-directed and GR-directed agents are also used in patients with ACTH-independent CS caused by an adrenal adenoma, adrenal hyperplasia or cortisol-producing adrenal carcinoma. In these patients, medical therapies are predominantly used to treat acute complications of CS and control hypercortisolism in advanced or recurrent disease (59).

This expanding therapeutic landscape allows for a patient-tailored approach in the treatment of CS and related comorbidities. Post-surgical normalization of hypercortisolaemia is generally associated with improved glucose metabolism, although insulin resistance and increased cardiovascular risk may persist in patients even after successful surgery (60, 61). Cortisol-lowering therapies along with antidiabetic medications can be used as effective adjunctive strategies in CD patients with glucose metabolism derangements who cannot undergo surgery or for whom surgical treatment was ineffective (19, 20). Medical treatments have various specific effects on glucose metabolism that are partially independent of their role in the normalization of hypercortisolaemia. These effects should be taken into consideration when making therapeutic decisions to provide adequate and patient-tailored treatment. Below, we summarize the clinical studies that investigated the effects of various cortisol-lowering therapies on glucose homeostasis.

## Pasireotide

Pasireotide is a new-generation somatostatin analog that binds four of the five known somatostatin receptors (SSTRs 1-3 and SSTR5) and demonstrates a significantly higher binding affinity for SSTR5 compared to octreotide. By stimulating SSTR5 and SSTR2, pasireotide activates downstream inhibitory signaling pathways, thereby suppressing hormone secretion, proliferation, and survival of pituitary gland cells (62). The efficacy of pasireotide in the treatment of CD was demonstrated in two multicenter phase III studies, and subsequently, it was approved by the Food & Drug Administration (FDA) as a first agent for patients with CD who are ineligible for surgical treatment or for whom surgery has failed (63, 64).

SSTR2 and SSTR5 receptors targeted by pasireotide are expressed by insulin-producing beta cells of the pancreas, while glucagon-secretin alpha cells express predominantly SSTR2 (62). Consequently, treatment with pasireotide has been associated with significant inhibition of insulin and incretins secretion, along with only a modest suppression of glucagon levels, which commonly leads to pasireotide-induced hyperglycemia (63–67). Indeed, phase II and III clinical trials demonstrated that treatment with short-acting pasireotide administered subcutaneously twice daily was associated with hyperglycemia and DM in 35-40% and 18-21% of patients, respectively. Hyperglycemia-related adverse events were observed in 73% of patients, and new antidiabetic medication was initiated in 45% of study participants. Notably, 41% of patients who had not been on any antidiabetic treatment required their first antidiabetic medication following treatment with pasireotide (64, 65). In the following phase III trial, once-monthly long-acting pasireotide demonstrated a similar safety profile. Hyperglycemia, DM, and hyperglycemia-related adverse effects were noted in 49%, 19%, and 72% of patients, respectively. Treatment with a new antidiabetic drug was started in approximately 50% of study participants (63).

Real-world evidence and clinical trial extension studies have demonstrated that glucose metabolism alterations tend to occur primarily within the first weeks of therapy with pasireotide and stabilize over time (68–71). The risk of developing pasireotide-induced hyperglycemia is the highest in patients with high glucagon levels, HbA1c > 34.5 mmol/L (5.3%), and glucose peak > 9 mmol/L after pasireotide administration (72). However, careful monitoring for glucose metabolism abnormalities is required for all patients undergoing therapy. The detailed considerations regarding the medical management of pasireotide-induced hyperglycemia in patients with CD are discussed later in this review.

## Cabergoline

Cabergoline is a dopamine D2 receptor agonist that has been commonly used in the treatment of prolactinomas (73). The expression of dopamine receptors was found in adrenocorticotrophic cells and ACTH-secreting pituitary adenomas, suggesting the potential efficacy of cabergoline in the therapy of CD (74, 75). The clinical efficacy of cabergoline was reported in case reports as well as several clinical studies that demonstrated normalization of urinary free cortisol (UFC) in 25-40% of CD patients (76–80). However, the recent prospective trial that involved 20 patients with CD failed to show gradual and dose-dependent correction of cortisol levels in CD patients treated with cabergoline (81). Although currently the clinical utility of cabergoline in the management of hypercortisolaemia remains debatable, the improvements in glucose metabolism associated with dopamine agonist therapy were frequently reported in patients with CD.

In a prospective open-label study, testing cabergoline at a dose of 1 mg/week, adjusted up to a maximal dose of 7 mg/week, showed a significant reduction of fasting serum glucose and insulin levels in CD patients responding to therapy (79). Furthermore, a retrospective multi-center study that analyzed cabergoline at a dose range of 0.5-6 mg/week as a first-line therapy or in persistent CD demonstrated improvement of glycemic control in 40% of responders (82). Notably, the improvement of glucose homeostasis was also observed in patients with persistent mean urinary free cortisol (mUFC) levels, which agrees with the previously reported impact of dopamine agonists on glucose metabolism that is independent of their cortisol-lowering effects (79). The direct mechanisms by which dopamine agonists improve glycemic control remain unclear, and apart from cortisol-lowering effects, they may involve the combined actions of dopamine agonists on the central nervous system, insulin secretion, and glucose uptake in the insulin-sensitive tissues (83).

## Ketoconazole and levoketoconazole

Ketoconazole is an azole antifungal drug that reduces adrenal steroid production by inhibiting various enzymes involved in steroidogenesis. It has been approved for the treatment of Cushing’s disease by the European Medicine Agency and is used off-label for this purpose in the USA (84). Although ketoconazole was shown to rapidly induce normalization of cortisol levels in CD patients, it can lead to hepatotoxicity that requires frequent monitoring in patients undergoing therapy (85). Furthermore, the clinical use of ketoconazole was limited by the long-standing lack of prospective studies evaluating its efficacy. Nevertheless, previously published retrospective studies suggested that ketoconazole might be effective in lowering HbA1c and fasting glucose in CD patients (86–88). In a large analysis, Castinetti et al. evaluated data on 200 CD patients treated with ketoconazole, from whom nearly 32% had DM at baseline. Notably, the authors observed improved glycemic control in more than half of diabetic patients (87). Another study examining 62 CD patients treated preoperatively with either ketoconazole, metyrapone, or their combination reported lowering of HbA1c levels in those patients whose cortisol levels were entirely or partially controlled by steroidogenesis inhibitors (88). These data suggest that ketoconazole can be considered for both short- and long-term therapy in patients with persistent CD and DM.

Levoketoconazole is the 2S, 4R enantiomer of ketoconazole. It inhibits adrenal steroid production more potently compared to ketoconazole and might also suppress ACTH secretion and proliferation of pituitary adenoma cells (89). Levoketoconazole was recently evaluated in two phase III prospective clinical trials in patients with endogenous CD (90, 91). Both studies reported sustained improvements in mUFC along with an acceptable safety and tolerability profile, which prompted the FDA approval of levoketoconazole for patients with CS ineligible for surgical treatment or for whom surgery has not been curative. In phase III, multicenter, open-label single-arm trial (SONICS), levoketoconazole was evaluated in three phases: dose titration (2-21 weeks to achieve effective and tolerable dose), maintenance phase (6 months of treatment at the therapeutic dose), and extended evaluation (6 months of continued treatment). The results of the first two phases showed that 81% of patients who advanced to the maintenance phase had mUFC normalization by end of dose titration, and at the end of the maintenance phase 31% of 94 patients enrolled in the study were responders. Importantly, levoketoconazole treatment led to significant improvements in biomarkers of CS comorbidities and glucose metabolism at the end of the maintenance phase, including fasting glucose concentration, HbA1c concentration, total and LDL cholesterol, and body weight (90). The efficacy of levoketoconazole was further analyzed *post-hoc* in patients with DM or without DM who entered the maintenance phase. In both groups, levoketoconazole treatment led to sustained normalization of mUFC and glycemic control, and the latter effect was most pronounced in patients with DM. The authors reported that at the end of the maintenance phase, HbA1c decreased from 6.9% at baseline to 6.2% and from 5.5% to 5.3% in patients with and without DM, respectively. Mean fasting blood glucose decreased from 6.85 mmol/L (123.4 mg/dL) to 5.82 mmol/L (104.9 mg/dL) in patients with DM and from 5.11 mmol/L (92.1 mg/dL) to 4.66 mmol/L (84 mg/dL) in patients without DM (92). In another phase III study (LOGICS), levoketoconazole was tested in patients with CD via an open-label titration maintenance phase, followed by a double-blinded randomized withdrawal phase and a restoration phase. The results from the interim analysis at the end of the randomized withdrawal phase demonstrated significantly better mUFC response and reduction of total and LDL cholesterol levels in patients with continuous levoketoconazole treatment. However, no significant differences in glycemic control markers between treatment groups were observed in this study (91). Thus, additional prospective trials and long-term analyses of the effects of levoketoconazole therapy on glucose homeostasis in CD patients are warranted.

Both ketoconazole and levoketoconazole are substrates and potent inhibitors of the cytochrome P450 (CYP3A4) enzyme, which is a major drug-metabolizing isozyme in the human liver. Therefore, drug-drug interactions should be considered when treatment with these agents is initiated. Medications that are major CYP3A4 substrates should be avoided in patients undergoing ketoconazole and levoketoconazole therapy (93).

## Metyrapone

Metyrapone reduces adrenal cortisol production by inhibiting the 11-beta-hydroxylase (CYP11B1), an enzyme that is responsible for the final step in the cortisol synthesis (94). Over the past decades, metyrapone has been extensively used as off-label therapy in the management of CD patients. Based on the evidence derived from observational and retrospective studies that demonstrated the clinical efficacy and safety of metyrapone, it was granted official approval by the European Medicines Agency for treating CS in 2014 (95). More recently, the prospective studies of metyrapone in patients with Cushing’s syndrome, including CD, were initiated to further determine its clinical utility and safety profile (96–98).

In an ongoing open-label, single-arm phase III/IV study (PROMPT), the efficacy of metyrapone is investigated in adults who were newly diagnosed with endogenous Cushing’s syndrome and had three baseline 24 hours urine free cortisol (UFC) values at least 50% above ULN (96). The starting dose of metyrapone is based on the severity of hypercortisolism at baseline and further titrated based on cortisol levels in urine and serum over 12 weeks (dose-titration period). After 12 weeks, patients whose mUFC do not exceed 2-fold the ULN continue to receive treatment for another 24 weeks (extension period). Early findings from this study, presented at the Annual Meeting of the Endocrine Society, indicated that mUFC normalization was achieved by 47% of patients and an additional 33% of patients had ≥ 50% decrease in mUFC from baseline. Circulating cholesterol, HbA1c and fasting glucose, and insulin improved with a median decrease of 12%, 3%, 5%, and 9%, respectively (96).

In a recent prospective, observational longitudinal study, Ceccato et al. analyzed 31 patients with CS treated with metyrapone for ≥ 1 month as primary treatment or after surgical failure. With a median dose of 1000 mg metyrapone for 9 months, UFC and late-night salivary cortisol (LNSC) decreased quickly after the first month of treatment (−67% and −57% from baseline), and sustained UFC normalization was observed up to 12 and 24 months (70% and 30% of patients had normalized UFC and LNSC at the last visit, respectively). Noteworthy, 7 out of 11 patients who presented with impaired fasting glucose or diabetes reduced the dose or the number of anti-diabetic drugs (97). In another study, the combination of mitotane, metyrapone, and ketoconazole initiated concomitantly led to rapid normalization of hypercortisolaemia along with the reduction of plasma fasting glucose and Hb1Ac levels in patients with severe CS (98). Retrospective analyses further confirm the utility of metyrapone in the rapid management of CS and cortisol-related comorbidities. Jeffcoate et al. and Verhelst et al. reported improvement of biomarkers of glucose metabolism in up to 80% of patients presenting with CS and glucose intolerance or DM (99, 100). However, the effects of metyrapone treatment on glucose metabolism were not analyzed in the largest retrospective study of 195 with Cushing’s syndrome, including 115 patients with CD (101).

Collectively, the current data suggest that metyrapone can be used to promptly normalize hypercortisolaemia and improve cortisol-related metabolic comorbidities in CD patients. Therefore, it may offer a useful treatment modality, either as a preoperative therapy or in the long-term management of patients with CD.

## Mitotane

Mitotane is an adrenolytic and adrenostatic agent currently used in the treatment of adrenocortical carcinoma and occasionally employed in the management of severe CS (102, 103). Several studies published in the last century reported the high efficacy of mitotane in the management of CS with an average remission rate ranging from 70 - 100%, albeit the observed response rates might have been partially attributable to the low reliability of the hormone assays available at that time (104–107). A retrospective analysis of 76 patients who were treated with mitotane between 1993 and 2009 demonstrated remission in 72% of cases, however, intolerance leading to treatment discontinuation was observed in nearly 30% of patients. Patients who achieved remission had significantly improved metabolic outcomes, including decreased levels of fasting and postprandial serum glucose (108). Although mitotane may provide effective control of hypercortisolaemia and cortisol-related comorbidities in patients not responding to other therapies, its clinical use is limited because of its poor safety profile. Moreover, there are no randomized clinical trials that evaluated the efficacy of mitotane in patients with CD.

## Osilodrostat

Osilodrostat is a potent inhibitor of 11-β-hydroxylase (CYP11B1) and aldosterone synthase (CYP11B2), which are the enzymes that catalyze the final steps of cortisol and aldosterone synthesis in adrenal glands (109). In recent years, several clinical studies demonstrated the efficacy of osilodrostat in the treatment of CD, which led to its FDA approval for patients who are not surgical candidates or who have persistent/recurrent disease after surgery (110–113). A multicenter phase III study (LINC 3) included a 24-week open-label, single-arm treatment with osilodrostat, followed by the randomized withdrawal phase for 8 weeks and open-label treatment with osilodrostat until week 48. The results of this trial demonstrated that at the end of the withdrawal phase, more patients maintained mUFC below the upper limit of normal (ULN) with osilodrostat compared to placebo. Moreover, the reduction of mUFC was accompanied by significant improvement in metabolic and cardiovascular-related parameters, including BMI, fasting plasma glucose, systolic and diastolic blood pressure, and total and LDL cholesterol. At week 48, the analysis of all patients enrolled in the study indicated that mean fasting plasma glucose decreased from 99.2 mg/dL at baseline to 87.2 mg/dL, while HbA1c decreased from 6.0% to 5.6% (110). The clinical efficacy and safety of osilodrostat were further confirmed in another phase III study that comprised of an initial 12-week, randomized, double-blind, placebo-controlled (osilodrostat: placebo, 2:1) period followed by a 36-week, open-label treatment period (LINC 4). In this trial, significantly more patients treated with osilodrostat than placebo achieved mUFC at 12 weeks (77% vs 8%), and the response was maintained at 36-week when 81% of all patients showed mUFC normalization. At week 12, major metabolic and cardiovascular-related parameters also showed improvement in osilodrostat, but not in the placebo group. Notably, in patients who were classified as diabetic at baseline, mean fasting plasma glucose decreased from 110.7 mg/dL at baseline to 101.8 mg/dL at week 12 and 98.2 mg/dL at week 48; mean HbA1c decreased from 6.7% at baseline to 6.3% at week 12 and 6.3% at week 48. In patients who were not classified as diabetic at baseline, fasting plasma glucose and HbA1c levels remained within the normal range and were stable throughout the treatment (113). These observations agreed with the previously published data from the phase II trial that also showed improvement in fasting glucose and HbA1c levels during osilodrostat treatment in patients with a history of DM (111).

Overall, osilodrostat is an effective medical therapy for patients with CD and has a significant potential to alleviate the burden of CD-related comorbidities, including insulin resistance and DM (114, 115).

## Mifepristone

Mifepristone is a high-affinity antagonist of the glucocorticoid receptor that affects both peripheral and central actions of cortisol, such as its negative feedback on the CRH/ACTH secretion (116, 117). Accumulating evidence suggests that mifepristone improves insulin sensitivity through its effects on many tissues and organs involved in the regulation of glucose homeostasis, although the underlying mechanisms are not fully understood (116). Recent clinical studies demonstrated the effectiveness of mifepristone in the management of clinical and metabolic features related to hypercortisolaemia. Consequently, it was approved by FDA for the treatment of CS with a specific indication for patients who have glucose intolerance or DM and for whom surgical treatment was not effective (118).

In 2012, Fleiseriu et al. reported the results from the largest prospective multicenter trial of mifepristone in the treatment of Cushing’s syndrome (SEISMIC). In this study, 50 patients with endogenous Cushing’s syndrome (including 43 CD patients) associated with DM/glucose intolerance (CS-DM, n=29) or hypertension (CS-HT, n=21) were recruited. Patients were treated with mifepristone at a dose of 300 mg-1200 mg/week for 24 weeks. In the CS-DM group, the area under the curve for glucose on 2h oral glucose test decreased by at least 25% in 60% of patients from baseline to end of therapy. Fasting plasma glucose and HbA1c decreased from 149.0 ± 74.7 mg/dL to 104.7 ± 37.5 mg/d and from 7.43 ± 1.52% to 6.29 ± 0.99%, respectively. Antidiabetic medications were reduced in 7 out of 15 patients and insulin daily dose was reduced by at least half in 5 out of 12 patients. Noteworthy, overall clinical improvement was seen in 87% of patients, and mifepristone therapy was associated with a significant reduction of body weight, waist circumference, and body fat, as well as increased insulin sensitivity (118–120). In a long-term extension and follow-up analysis of the SEISMIC study, clinically meaningful weight loss persisted for two additional years in patients who remained on the mifepristone therapy (121).

The European, multicenter, retrospective study on 20 patients with CS (4 patients with CD, 15 patients with malignant disease due to adrenocortical carcinoma or ectopic ACTH secretion) treated with mifepristone at doses of 600-1200 mg/day reported improvement of clinical features in 75% of cases. Normalization of glucose control was observed in 4 out of 7 patients, which further suggests that mifepristone may effectively improve glycemic control in patients with hypercortisolaemia (122).

Taken together, mifepristone appears to be an effective and well-tolerated therapeutic option for patients with CD and diabetes mellitus or impaired glucose tolerance. Nevertheless, the clinical use of mifepristone requires close monitoring of severe adverse effects, including hypokalemia. Due to its abortifacient properties mifepristone must be used with caution in women of childbearing age.

## Relacorilant

Relacorilant is an investigational selective glucocorticoid receptor (GR) modulator. Contrary to mifepristone, relacorilant lacks the affinity for the progesterone receptor. Thus, relacorilant limits cortisol activity without undesirable side effects related to progesterone receptor antagonisms, such as abortive properties and irregular vaginal bleeding (118, 123). In 2021, Pivonello et al. reported the efficacy and safety of relacorilant in a single-arm, open-label, phase 2 study which enrolled 35 patients with a diagnosis of endogenous CS and concurrent uncontrolled hypertension and/or impaired glucose tolerance or DM (124). In this study, relacorilant was administered at low dose (100-200 mg/d) for 12 weeks or high-dose (250-400 mg/d) for 16 weeks. Among patients with hyperglycemia, clinically meaningful hyperglycemia response (defined *ad-hoc* as ≥ 0.5% decrease in HbA1c, normalization or ≥ 50mg/dl decrease in 2h plasma glucose on OGTT or decrease in daily insulin or sulfonylurea dose by 25% and 50%, respectively) was observed in 2 of the 13 patients in the low-dose group and 6 of the 12 patients in the high-dose group. Common adverse effects included back pain, headache, peripheral edema, nausea, pain in the extremities, dizziness, and diarrhea. No clinically significant hypokalemia was observed.

Currently, two randomized, double-blind, placebo-controlled study phase III clinical trials are conducted to further evaluate the efficacy and safety of relacorilant in patients with CS (GRACE, NCT03697109 and GRADIENT, NCT04308590).

## Antidiabetic treatment in patients with Cushing’s disease

Antidiabetic treatment plays an essential role in the management of patients with hypercortisolaemia and concurrent DM. Antidiabetic medications are often combined with cortisol-lowering agents to achieve glycemic control in patients with persistent or recurrent disease after surgical treatment. Nevertheless, the clinical evidence regarding the optimal antidiabetic treatment in patients with CD is scarce, and the current recommendations are largely based on expert opinions and algorithms used for type 2 DM.

*Metformin* in combination with cortisol-lowering agents is considered first-line therapy in the chronic management of CD patients with persistent hypercortisolaemia and hyperglycemia (15). Metformin reduces circulating glucose levels by lowering hepatic glucose production and improving peripheral insulin sensitivity, and therefore it may effectively control symptoms of hyperglycemia and reduce long-term HbA1c levels in CD patients. Metformin is also considered a first-line treatment in patients with pasireotide-induced hyperglycemia (68, 125, 126). Treatment with metformin is generally safe and well tolerated, however, it may cause undesired gastrointestinal effects, which can be potentiated by concomitant use of cortisol-lowering medications, such as pasireotide and osilodrostat (62, 115).

*Incretin-based therapies with Glucagon-like peptide 1 (GLP-1) receptor agonists and dipeptidyl peptidase-4 (DPP-4) inhibitors* can be considered second-line treatments, and they are usually combined with metformin in patients who need treatment intensification (80). GLP-1 agonists reduce hyperglycemia by enhancing insulin secretion and inhibiting the production of glucagon, and they delay gastric emptying and reduce appetite (127). The positive effects associated with GLP-1 agonists therapy include weight loss and blood pressure reduction (128). DPP-4 inhibitors block the degradation of GLP-1 and GIP, thereby increasing their endogenous levels. This in turn leads to increased insulin secretion and reduced postprandial and fasting hyperglycemia (127, 129). DPP-4 inhibitors do not have significant cardiovascular benefits and are weight-neutral (130). Because the incretin-based treatments are generally well-tolerated and reduce postprandial glycemia, they provide useful therapeutic options in the management of patients with CD and concurrent DM (131).

*Sodium-glucose cotransporter 2 (SGLT-2) inhibitors* were demonstrated to reduce cardiovascular risk and may have positive effects in patients with heart failure and impaired renal function (132). Nevertheless, SGLT-2 inhibitors increase the risk of genitourinary infection, and therefore they should be used with caution in patients with CD who may be prone to serious infection and systemic dissemination (133). *Sulfonylureas and glinides* can be recommended for short-term periods to manage postprandial glycemia, although these agents increase the risk of hypoglycemia and are rarely used independently in the long-term management (134). *Peroxisome proliferator-activated receptor-γ (PPARγ) agonists* improve insulin sensitivity in the liver and skeletal muscles, however, they often cause weight gain and edema, and therefore are generally not recommended in patients with CD (135).

*Insulin* therapy may be required when glycemic control cannot be achieved with other agents. In these cases, the combination of metformin with long-acting basal insulin analog is usually initiated as a first option, and the addition of prandial insulin should be considered in patients who present with poor glycemic control and/or high post-prandial glucose levels. In patients with severe disease who demand prompt management of hyperglycemia, a combination of insulin treatment in the form of an infusion, prandial insulin, or basal-bolus regimens combined with cortisol-lowering therapies may be needed to rapidly achieve glycemic control (15).

Currently, the development of the optimal antidiabetic strategies for patients with CD is limited by the lack of clinical studies evaluating the efficacy of different therapeutic options in that population. Given the growing role of medical therapies in the management of patients with CD, there is also an unmet need to identify the most effective and safe combinations of antidiabetic agents and cortisol-lowering medications.

## Management of hyperglycemia induced by pasireotide treatment

All patients treated with pasireotide should be carefully monitored for the development of impaired glucose tolerance and diabetes mellitus (62, 68). Although the optimal treatment for managing pasireotide-induced hyperglycemia is not well-established, metformin is usually considered a first-line therapy. If adequate glycemic control is not achieved with metformin alone, combination treatment with incretin-based therapies can be initiated. DPP-4 inhibitors and GLP-1 analogs may be effective in the management of pasireotide-induced hyperglycemia, as pasireotide impairs both pancreatic insulin secretion and incretin response. The staged treatment intensification with DPP-4 inhibitor with a subsequent switch to a GLP-1 receptor agonist was suggested by expert recommendations (136). GLP-1 analogs may provide additional advantages compared to DPP-4 inhibitors in the management of pasireotide-induced hyperglycemia as they demonstrated the potential to reduce body weight and have a superior HbA1c lowering effect. Furthermore, inhibition of GLP-1 degradation by DPP-4 inhibitors may not effectively restore GLP-1 levels when its secretion has been already impaired by pasireotide (137). Nevertheless, further studies are needed to determine the optimal order and regimen of incretin-based therapies in the management of pasireotide-induced hyperglycemia. If hyperglycemia induced by pasireotide treatment remains uncontrolled by the combinations of metformin and incretin-based therapies, the initiation of insulin therapy is required to achieve and maintain glycemic control (138).

## Conclusions

Impairment of glucose metabolism is one of the most common complications encountered in patients with CD. Further research aiming to elucidate the pathogenesis of glucose intolerance and DM in patients with hypercortisolaemia is warranted and may provide novel therapeutic opportunities. With the exception of pasireotide, new medical therapies were demonstrated to improve glucose intolerance and glycemic control. The ongoing and future clinical studies should aim to identify the optimal combinations of antidiabetic medications and cortisol-lowering therapies for the tailored treatment of CD patients with concurrent DM. There is also a need to facilitate the selection of patients who may benefit from specific combination regimens.

## Author contributions

AM conceptualization, literature analysis, writing and editing of the manuscript. MB literature analysis, editing of the manuscript. DM literature analysis, writing and editing of the manuscript. PW conceptualization, literature analysis, writing and editing of the manuscript, supervision. All authors contributed to the article and approved the submitted version.
